# Leveraging high-throughput screening data, deep neural networks, and conditional generative adversarial networks to advance predictive toxicology

**DOI:** 10.1371/journal.pcbi.1009135

**Published:** 2021-07-02

**Authors:** Adrian J. Green, Martin J. Mohlenkamp, Jhuma Das, Meenal Chaudhari, Lisa Truong, Robyn L. Tanguay, David M. Reif

**Affiliations:** 1 Department of Biological Sciences, and the Bioinformatics Research Center, NC State University, Raleigh, North Carolina, United States of America; 2 Department of Mathematics, Ohio University, Athens, Ohio, United States of America; 3 Marsico Lung Institute, University of North Carolina at Chapel Hill, Chapel Hill, North Carolina, United States of America; 4 Department of Computational Science and Engineering, North Carolina A&T State University, Greensboro, North Carolina, United States of America; 5 Department of Environmental and Molecular Toxicology, Oregon State University, Corvallis, Oregon, United States of America; Ecole Polytechnique Fédérale de Lausanne, SWITZERLAND

## Abstract

There are currently 85,000 chemicals registered with the Environmental Protection Agency (EPA) under the Toxic Substances Control Act, but only a small fraction have measured toxicological data. To address this gap, high-throughput screening (HTS) and computational methods are vital. As part of one such HTS effort, embryonic zebrafish were used to examine a suite of morphological and mortality endpoints at six concentrations from over 1,000 unique chemicals found in the ToxCast library (phase 1 and 2). We hypothesized that by using a conditional generative adversarial network (cGAN) or deep neural networks (DNN), and leveraging this large set of toxicity data we could efficiently predict toxic outcomes of untested chemicals. Utilizing a novel method in this space, we converted the 3D structural information into a weighted set of points while retaining all information about the structure. *In vivo* toxicity and chemical data were used to train two neural network generators. The first was a DNN (Go-ZT) while the second utilized cGAN architecture (GAN-ZT) to train generators to produce toxicity data. Our results showed that Go-ZT significantly outperformed the cGAN, support vector machine, random forest and multilayer perceptron models in cross-validation, and when tested against an external test dataset. By combining both Go-ZT and GAN-ZT, our consensus model improved the SE, SP, PPV, and Kappa, to 71.4%, 95.9%, 71.4% and 0.673, respectively, resulting in an area under the receiver operating characteristic (AUROC) of 0.837. Considering their potential use as prescreening tools, these models could provide *in vivo* toxicity predictions and insight into the hundreds of thousands of untested chemicals to prioritize compounds for HT testing.

## Author summary

A combined deep neural network (DNN) and conditional Generative Adversarial Network (cGAN) can leverage a large chemical set of experimental toxicity data plus chemical structure information to predict the toxicity of untested compounds.

## Introduction

Currently, there are 85,000 chemicals registered with the EPA, as part of the Toxic Substances Control Act[1], that are manufactured, processed, or imported into the United States; however, only 4,400 have rigorous toxicological data, leaving over 80,000 chemicals untested [2,3]. Due to the high cost, and ethical concerns over the use of low-throughput mammalian models associated with traditional *in vitro* and *in vivo* assays, there has been increasing demand to reduce the number of animals used in toxicity testing paradigms by switching to *in silico* methods [4]. To directly address this chemical data gap and help prioritize chemicals for testing, both computational and high-throughput screening (HTS) approaches have been employed. The EPA developed the ToxCast program, an HTS approach, which included approximately 700 biochemical and cell-based assays, which was efficient but lacking in systemic biological complexity [2,5,6]. Therefore, as part of an effort to expand the toxicology database, a multidimensional HTS assay was devised to examine all ToxCast phase 1 and 2 chemicals (over 1,000 unique chemicals) for developmental- and neuro-toxicity in the embryonic zebrafish [7]. While computational approaches to bridge the data gap above have been developed, with Quantitative Structure-Activity Relationship (QSAR) and Read-Across being the most commonly used methodologies [8–13]. Both methods rely on the grouping of chemicals together using fragment descriptors, e.g. number of carbons, types of bonds, functional groups, etc. and have employed statistical or machine learning approaches [14–16]. Although these methods have been useful in identifying priority compounds for further testing, how these chemicals are grouped together might add bias, and recent machine learning advances have not been thoroughly explored [14,17].

Machine learning is a method of data analysis that automates the building of analytical models [18]. It is a branch of artificial intelligence based on the idea that systems can learn from data, identify patterns, and make decisions[19,20]. It encompasses a very broad range of supervised (minimal human intervention) and unsupervised (no human intervention) algorithms. Generally developed for computer science, sophisticated nonlinear machine learning algorithms have been increasingly used in cheminformatics and predictive toxicology with support vector machines (SVM), random forest (RF), deep neural networks (DNN), and Bayesian based methods being the most widely used [9,16,21–33]. More recently, GANs have gained prominence, where two neural networks (generator vs discriminator) are pitted against each other to generate a data distribution similar to the input [34,35]. This methodology was successfully used to design *de novo* molecules with desired properties in drug discovery and photovoltaic material design [36–39]. GANs can be extended to a conditional model (cGAN) if both the generator and discriminator are trained using some extra information, in this case a unique identifier.

Although GANs have been used to design new molecules, to our knowledge no research has been done to investigate their utility in predictive toxicology. Considering that tens of thousands of chemicals are manufactured or imported into the United States annually without rigorous toxicity data, it is imperative that new, structure-based models are developed to predict toxicity for priority testing. Therefore, the objective of this project is to use DNN and cGAN to leverage the zebrafish HTS assay data along with chemical structure information to predict the toxic outcomes of untested chemicals.

## Materials and methods

**Fig 1:**
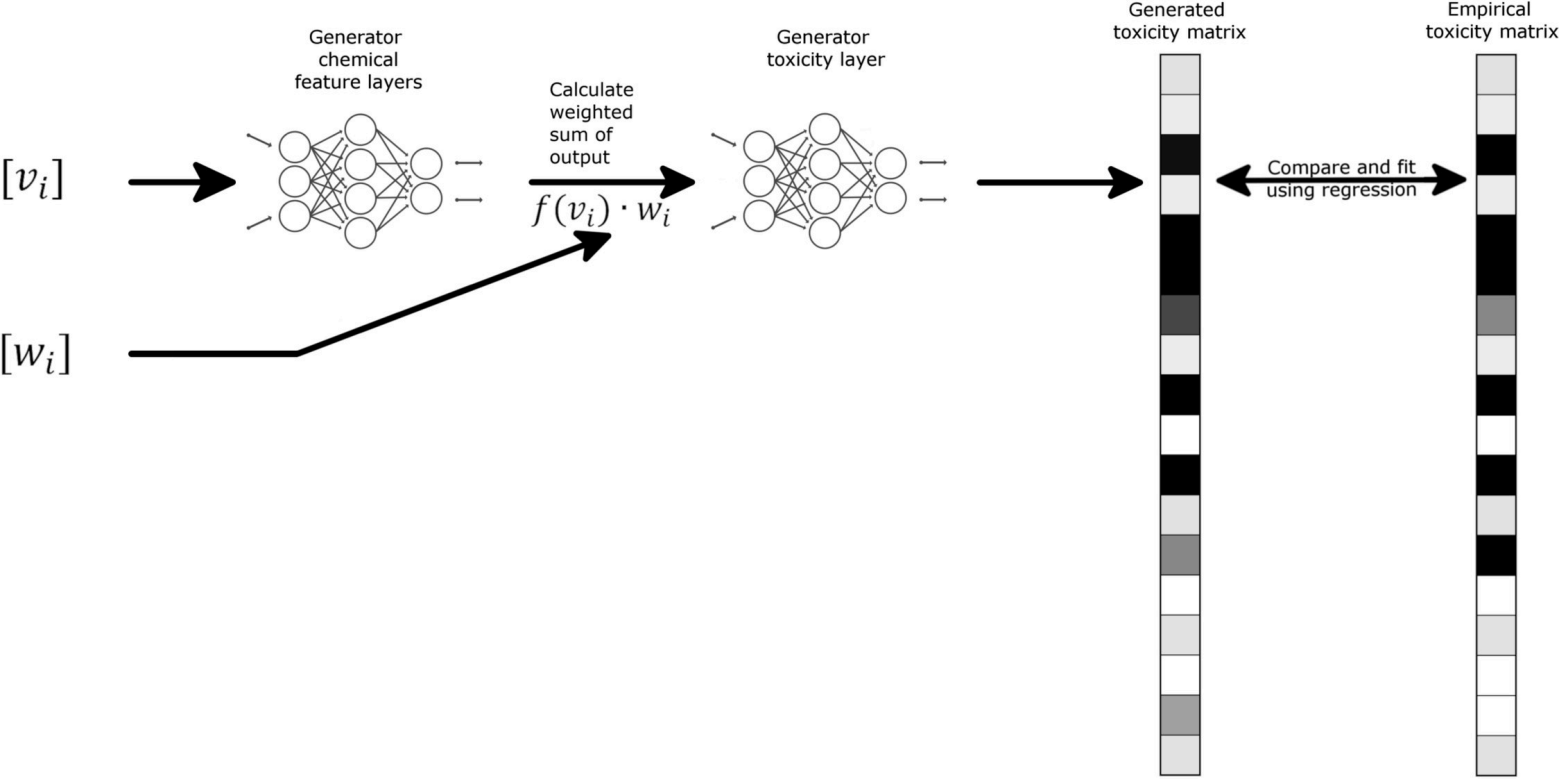
Regression generator diagram. Schematic representation of Go-ZT architecture showing chemical structural input represented as weights (w_*i*_) and views (v_*i*_) matrices passed through two fully connected neural networks to produce a predicted toxicity matrix. Darker matrix shading indicates higher toxicity values.

**Fig 2:**
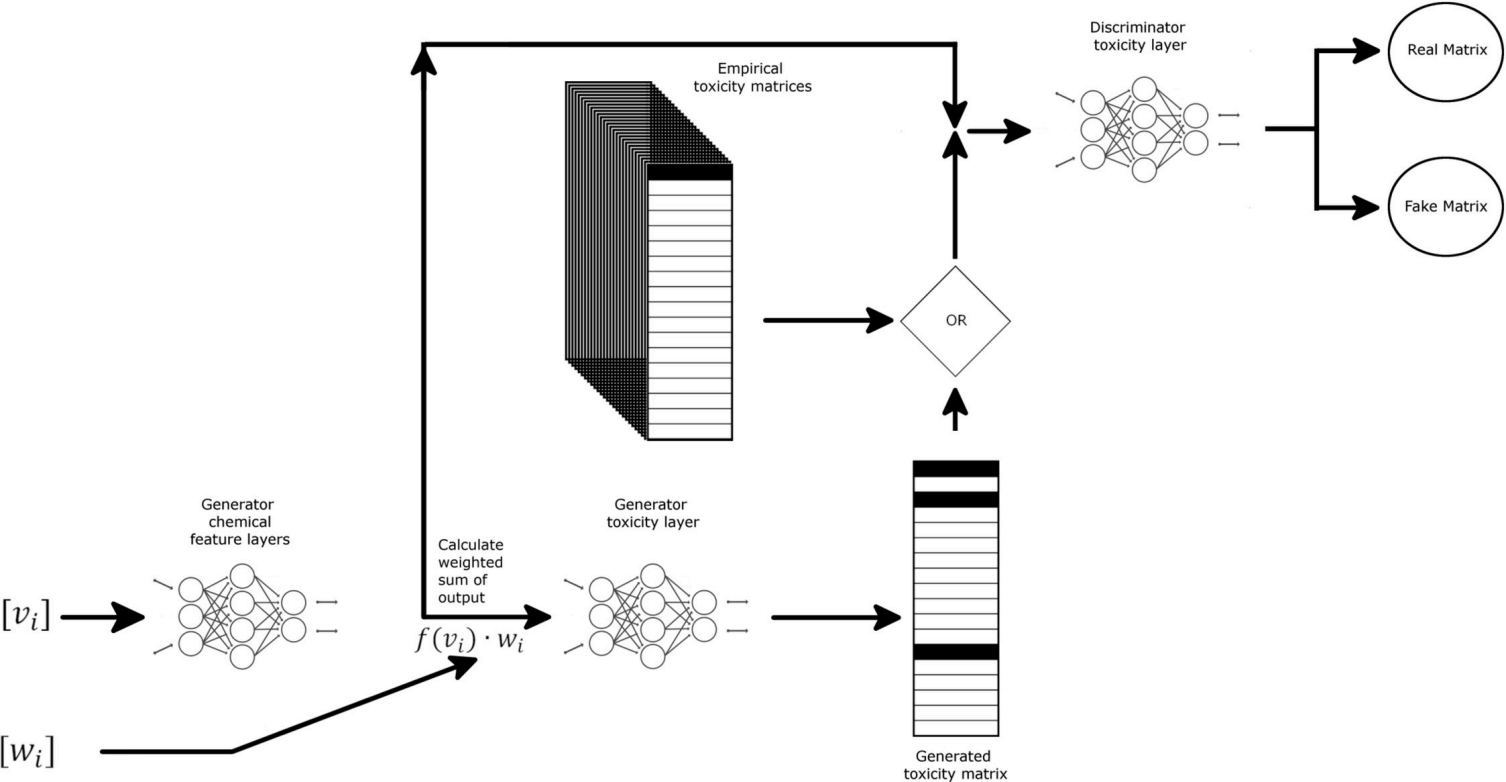
Conditional GAN diagram. Schematic representation of GAN-ZT architecture showing chemical structural input represented as weights (w_*i*_) and views (v_*i*_) matrices passed through two fully connected neural networks to produce a predicted toxicity matrix. Chemical features along with predicted or empirical toxicity matrices are then passed to a discriminator comprising a fully-connected neural network. Darker matrix shading indicates higher toxicity values.

**Fig 3:**
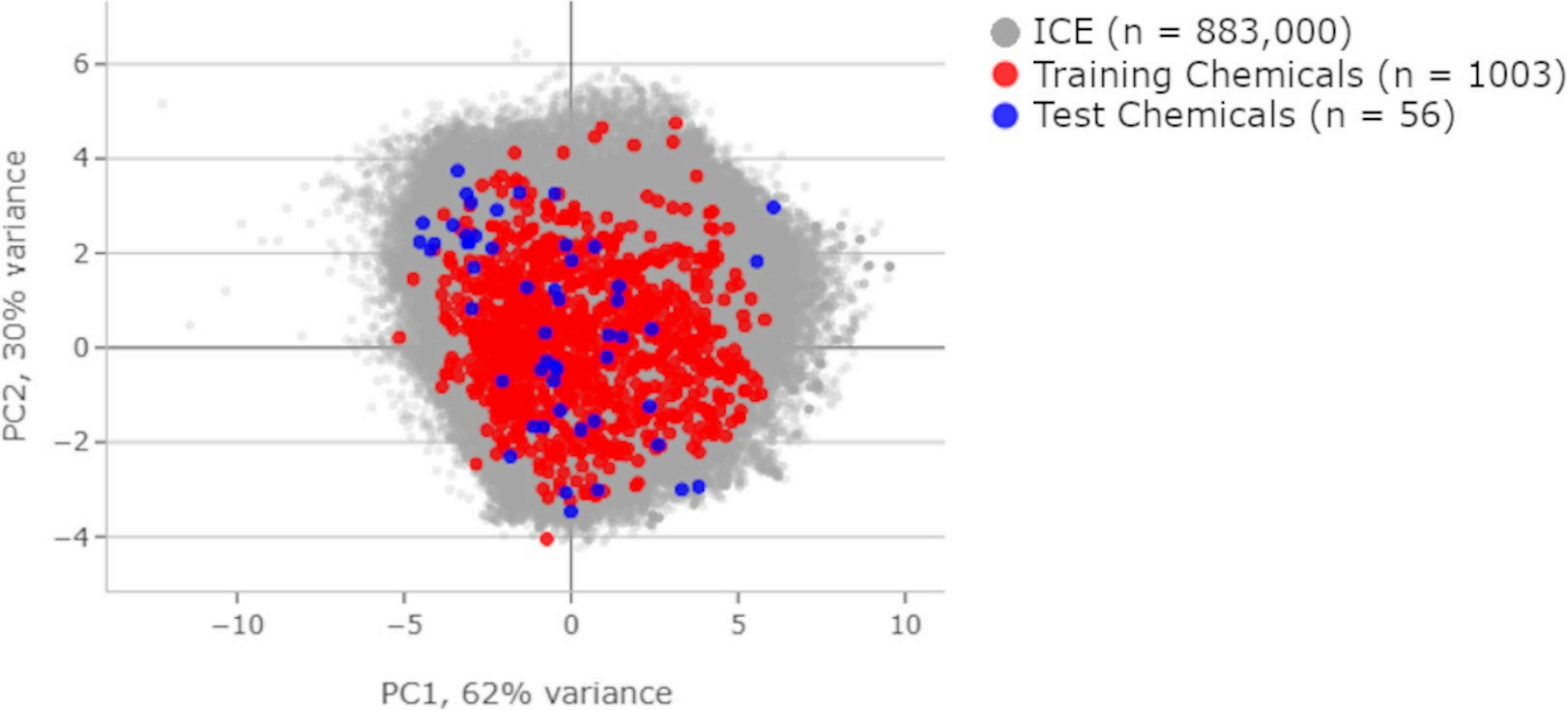
Data subdivision. Principal component analysis displayed against the background of over 800,000 chemicals in the Integrated Chemical Environment database. Compares physical chemical properties between the training and test sets.

In this section, we describe a cGAN and DNN utilizing a novel 3D molecular vectorization algorithm to predict active developmental toxicants. An overview of our approach is shown in Figs 1 and 2. First, we used experimental data collected on a large, diverse compound set to assess the toxic effects of these chemicals following developmental exposure (Fig 3). Next, we recast the chemical data in a structural representation that maintained connectivity and positional information of each atom in the molecule but in a format easily read as input into a neural network. Next, we trained two types of generators to produce toxicity data using the recast chemical structural representation. The first used a deep neural network (DNN) with regression (Fig 1) while the second utilized a cGAN architecture for training (Fig 2). This was done to produce generators capable of predicting developmental zebrafish toxicity data dependent on chemical structure alone. Regression training was leveraged to maximize model fit, minimize training time, and utilized a simpler toxicity data representation. cGAN training minimized the effects of outliers and increased network adaptability to chemical structure. All feature layers and toxicity layers shown in Figs 1 and 2 are DNN’s. These generators were trained using phase 1 and 2 ToxCast chemical data (n = 1003) split 80:20 into training and validation sets (Fig 3). Finally, we evaluated the trained networks on an independent test set containing chemicals (n = 56) of greater diversity in terms of both size and atomic constituents (Fig 3). These data were collected as part of an ongoing follow-up screen.

### Empirical (experimental) data

**Fig 4:**
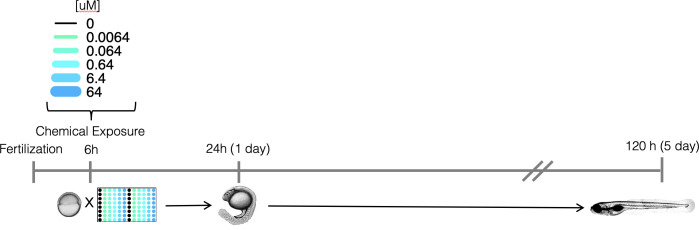
Experimental design. Schematic representation of the experimental approach for screening developmental and neurotoxicity of chemicals in larval zebrafish.

The empirical data used to develop a generator of zebrafish toxicity were gathered as described in Truong et al. and Noyes et al.[7,40]. Fig 4 shows the experimental design. The data included 1003 unique ToxCast chemicals tested at six concentrations for each chemical (0 μM, 0.0064 μM,0.064 μM, 0.64 μM, 6.4 μM and 64 μM). To minimize effects of response variability at lower concentrations, only the highest concentration was chosen for network training. There were 32 replicates (an individual embryo in singular wells of a 96-well plate) at each concentration for each chemical. At 120 hours post-fertilization (hpf), 18 distinct developmental endpoints were evaluated. The data were recorded as binary incidences and used to develop machine-learning models.

In a similar manner, toxicity matrices were created for an independent external test set of 56 chemicals that were collected in new experiments after collection of the original ToxCast data. This new test chemical set was more diverse in terms of atomic species and physical chemical properties (Fig 3)[41]. Due to chemical vectorization constraints we defined a reasonable domain of applicability to exclude, Perfluorinated chemicals with carbon chains longer than nine, Chloroperfluoro chemicals, and chemicals with a betaine functional group. Fig 3 shows the division of these data into train/test subsets and Principal Component Analysis (PCA) comparisons of the physical chemical properties using the Integrated Chemical Environment chemical characterization tool to highlight the diversity of the chemical domain space [41]. The PCA analysis includes the following physical chemical properties: Molecular Weight, Boiling Point, Henry’s Law, Constant Melting Point, Negative Log of Acid Dissociation Constant, Octanol-Air Partition Coefficient, Octanol-Water Distribution Coefficient, Octanol-Water Partition Coefficient, Vapor Pressure, and Water Solubility[41].

### Representing chemical compounds

**Fig 5:**
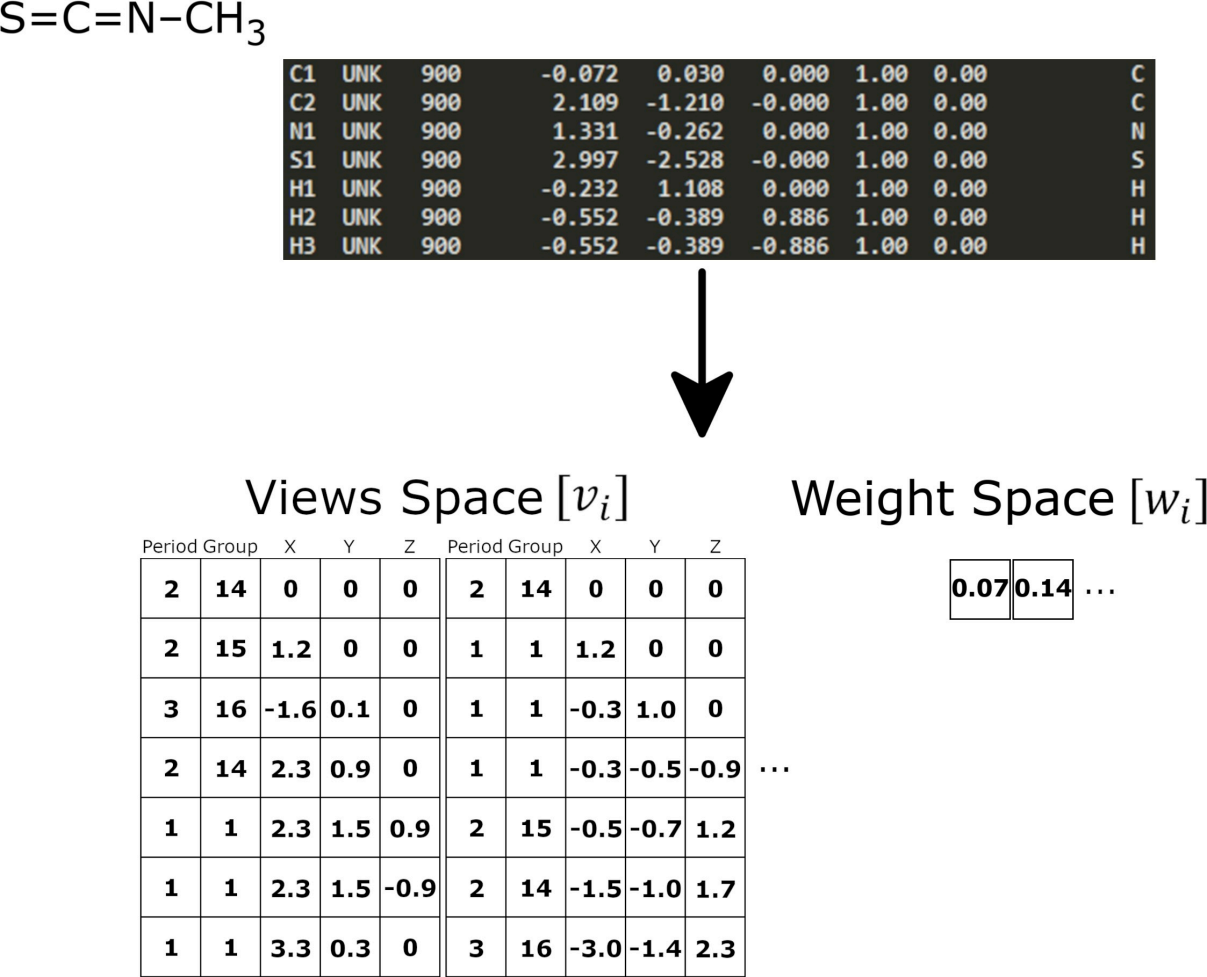
Diagram showing the vectorization of Methyl isothiocyanate. Atom information from the PDB file (shown in grey) in converted into the views and weights matrices. The views space (v_*i*_) columns one and two identify the chemical species and correspond to an atom’s position on the periodic table indicating their period and group, respectively. While the last three columns show the relative position of each atom. The weight space (w_*i*_) values correspond to each of the views space matrices. In the first views Table C1 is set at the center while in the second view C2 is set at the center of the view. This molecule has nine views, which can be reduced to three views if preference is given only to carbon.

The molecular structure of a chemical can be described or represented in various levels of complexity including molecular formula (1D), two-dimensional structural formula (2D), and three-dimensional, conformation-dependent (3D) with 2D being the most popular among chemists [42]. All three methods have been used to encode this structural information for utilization in deep learning, including, chemical properties, molecular fingerprints, SMILES, and graph vectorization, as well as 2D images of a chemical [38,43,44]. Considering that 2D representations are the most popular, ToxPrints, a molecular fingerprinting method will be used for benchmark evaluation. Utilizing CAS numbers chemical structural information and ToxPrints were retrieved from the EPA’s Chemistry Dashboard [45]. The structural information was converted from SDF to PDB format using Open Babel [46]. The PDB format was chosen as it is easily accessible and contains 3D structural information for all atoms in a molecule. Though a number of quantum chemistry based methods and software packages are available for 3D molecular vectorization [42], in this analysis, we utilized an novel algorithm developed to map and vectorize structure that was originally created for use in material sciences [47]. The PDB file for each chemical was vectorized as described by d’Avazac et al. [47] and illustrated in Fig 5. This method is simple and universal with few parameters and was adapted as follows: a view was started from each atom, or, by user option, only from each carbon atom (when available).

Onto each view, the remaining atoms (up to a user-defined limit) were added in order of their distance from the first atom; ties were broken by spitting a view into two or more views and the origin and orientation were determined by canonical rules. Lastly, an atom’s position on the periodic table was used as a unique identifier (group and period), together with location (x, y, and z coordinates) producing five chemical features per atom.

### Network performance and evaluation

Previous work analyzing this data has shown that a summary value, aggregate entropy (AggE) can be calculated from the 18 morphological endpoints[48]. The intent of this summary value is to capture a meaningful measure of toxicity, while avoiding overinflation by summing highly correlated endpoints. Using the threshold value (9.35) identified by Zhang et al. for AggE in these data, compounds may be classified as active or inactive[48]. It should be noted that this threshold value influences the toxicity hit rate of a chemical and is concentration dependent. Therefore, it would need to be changed when investigating other nominal concentrations. Following training, the resulting generators were used to output toxicity matrices. AggE values were calculated using both the empirical and generated toxicity matrices and compounds were classified as active or inactive using the threshold value identified above. Active vs inactive classification accuracy was evaluated using a confusion matrix, Cohen’s Kappa statistic, and area under the receiver operating characteristic (AUROC) as Kappa and AUROC measure model accuracy, while compensating for simple chance[49]. The primary metrics we used from the confusion matrix included sensitivity (SE), specificity (SP), and positive predictive value (PPV) as these parameters give us the true positive rate, true negative rate, and the proportion of true positives amongst all positive calls[50–52]. The network with the highest Kappa statistic and positive predictive value (PPV) was used for evaluation of the test dataset.

### Data imbalance

All datasets showed strong active vs inactive class imbalance (Table 1). Classifiers may be biased towards the major class (inactive) and, therefore, show poor performance accuracy for the minor class (active) [53]. To address this problem, we used Cohens Kappa statistic and positive predictive value (PPV) to evaluate model performance.

**Table 1:** Summary of training and testing data used in this study.

Data	Active Chemical	Inactive Chemical	Total
Training data	159	844	1003
External testing data	7	49	56

### cGAN and regression generator

Two network architectures were developed and tested to train a generator that was capable of using 3D chemical information, in the form of vectorize views (Fig 5), and generate a toxicity matrix (Figs 1 and 2). The following two different models were trained on a Dell R740 containing two Intel Xeon processors with 18 cores per processor, 512 GB RAM, and a Tesla-V100-PCIE (31.7 GB) using the open source Python library Keras [54] on top of TensorFlow as the backend [55] within a purpose build Singularity container environment [56]. Swish activation for hidden and output layers, batch normalization between layers, mean squared error for the loss function, stochastic gradient descent (SGD) as the generator optimizer, Adam as the discriminator optimizer, a kernel initializer with a Gaussian distribution, and without dropout were used [57–60]. For each model, we optimized the hyperparameters (i.e, the number of hidden layers, the number of nodes in the layers, the number of chemical views, number of atoms per view, loss functions, optimizers, learning rates etc.) by random search technique followed by a 10-fold cross-validation using a randomized 80:20 split using Cohens Kappa statistic as the objective metric.

The first model was a simpler deep neural network trained to produce a toxicity matrix using regression (Fig 1). We used multiple layers consisting of a deep neural network base layer to extract salient features from the views matrix for each chemical structure (generator chemical feature layer). A second layer calculated the weighted sum over features (*f*(*v_i_*)∙*w_i_*) and a final deep neural network (generator toxicity layer) generates toxicity values. The regression generator (Go-ZT) was trained over the course of 75 epochs (7 seconds/fold).

**Fig 6:**
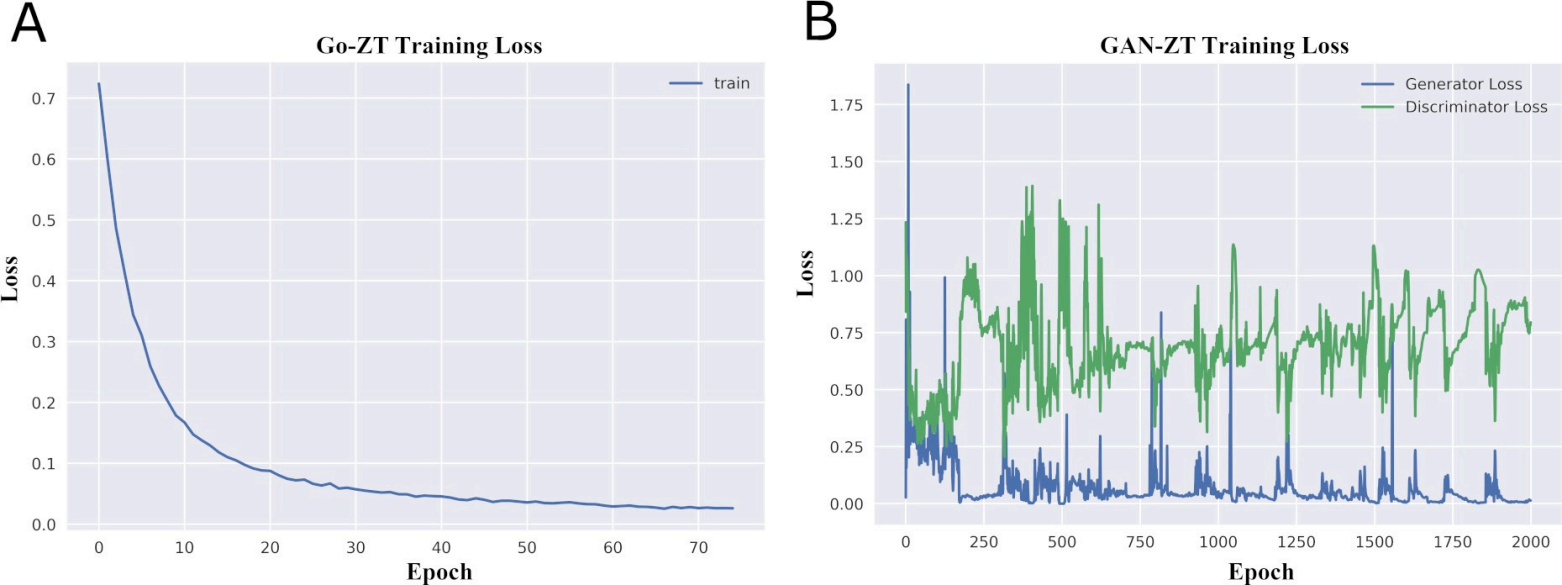
Go-ZT and GAN-ZT loss functions during training. Changes of loss functions during the training of (A) Go-ZT and (B) GAN-ZT.

The second model developed (GAN-ZT) used a much more complex cGAN architecture to generate a toxicity matrix (Fig 2). Similar to the Go-ZT above, we used multiple layers consisting of a deep neural network base layer to extract salient features from the views matrix for each chemical structure (generator chemical feature layer) and a second layer to calculate the weighted sum over these features (*f*(*v_i_*)∙*w_i_*). The resulting weighted sum of views (*f*(*v_i_*)∙*w_i_*) for each chemical was used as input to a final deep neural network (generator toxicity layer) to produce a generated toxicity matrix. The discriminator took the generators resulting weighted sum of views (*f*(*v_i_*)∙*w_i_*) for each chemical along with its corresponding empirical or generated toxicity matrices to determine whether the toxicity matrix was real or fake. This information was then backpropagated to train the generator. GAN-ZT was trained over the course of 2000 epochs (2 hours/fold). Fig 6 shows the training loss for both Go-ZT and GAN-ZT.

### Other classifiers

To evaluate the performance of the deep-learning and cGAN models in relation to other methods and chemical representation, we constructed the support vector machines (SVM), multilayer perceptron (MLP), and random forest (RF) models using KNIME (version 4.3.2) [61], and EPA ToxPrints, respectively [45]. We optimized the hyperparameters by a randomized search technique with cross-validation, using Cohens Kappa as the objective metric.

## Results

### Performance of five machine learning algorithms using cross-validation

Empirical and generated toxicity matrices for each chemical from the training and test datasets (Fig 3) were used to calculate AggE values to determine activity classification. The empirical training dataset contains 159 chemicals that meet the AggE threshold of 9.35 to be classified as an active compound (15.9%), as shown in Table 1. Table 2 shows the mean and standard deviation for the five machine learning algorithms trained using a 10-fold cross-validation. Go-ZT and GAN-ZT outperformed SVM, MLP, and RF using Kappa as the primary measure of performance. The GAN-ZT, SVM, MLP, and RF models showed poor performance while Go-ZT achieved moderate predictive performance and a good PPV percentage.

**Table 2:** Performance of different methods in activity classification with 10-fold cross-validation.

Model	SE		SP		PPV		Kappa		AUROC	
	Mean	SD	Mean	SD	Mean	SD	Mean	SD	Mean	SD
SVM	17.7	10.9	92.1	3.6	0.30	15.7	0.115	0.13	0.410	0.10
MLP	12.2	7.60	94.5	1.6	28.2	14.3	0.085	0.10	0.607	0.07
RF	6.5	6.10	**98.2**	3.1	56.7	44.1	0.071	0.09	0.609	0.08
GAN-ZT	**58.4**	20.7	64.1	19.4	28.4	5.4	0.160	0.05	0.613	0.03
Go-ZT	44.6	7.25	97.1	1.65	**76.1**	10.2	**0.495**	0.08	**0.709**	0.04

### External validation using independent test data

**Fig 7:**
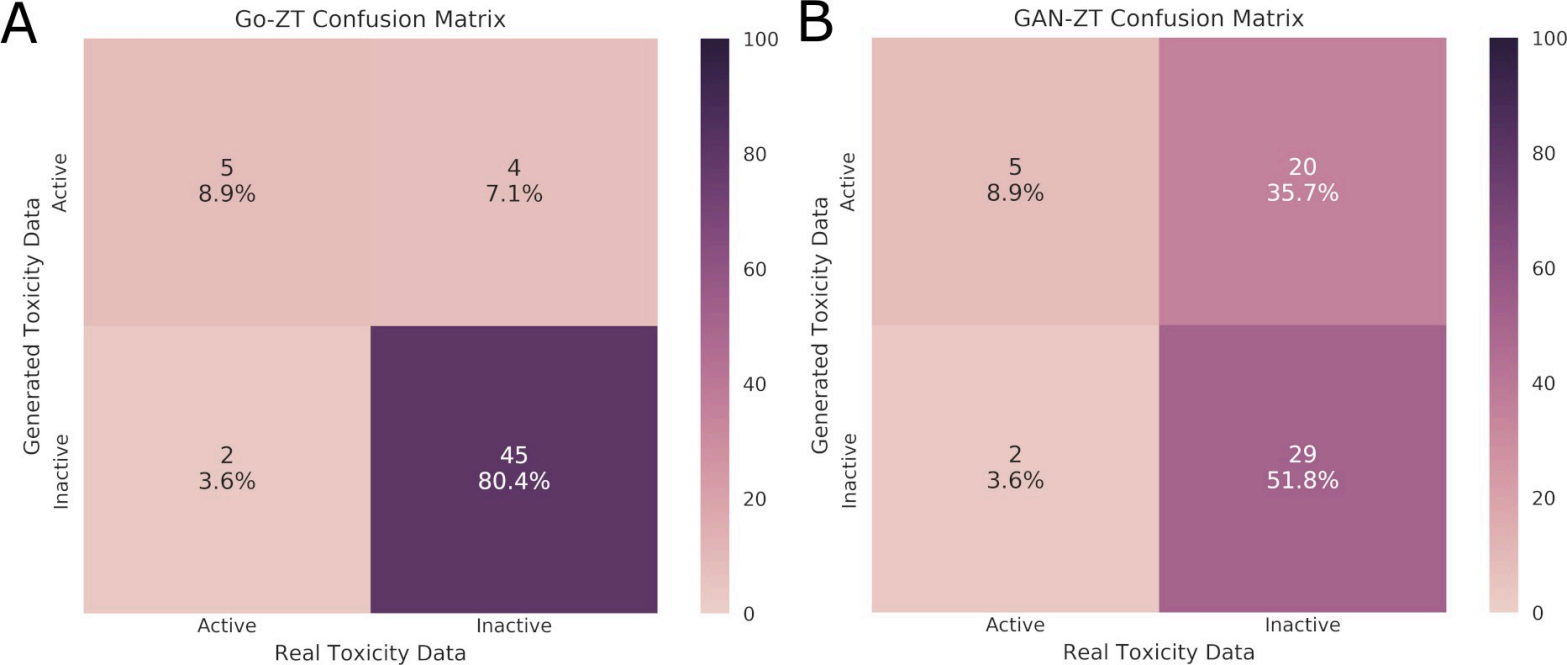
Test dataset confusion matrices. Evaluation of the classification of chemicals in the test data set as either active or inactive using real versus generated toxicity matrices by (A) Go-ZT or (B) GAN-ZT. Color scale represents percent of total chemicals.

We built final models using all training data and their best parameters, following hyperparameter optimization, and assessed their performance using an external testing dataset containing 7 active compounds (12.5%) as shown in Table 1. Considering the very slow pace of GAN-ZT training we used the same number of chemical features, number of views, number of atoms per view, and number of hidden layers for the generator chemical feature layers (three), and generator toxicity layers (11) for both GAN-ZT and Go-ZT (Fig 1 and 2). As shown in Table 3, four of the five models showed improved performance on the test set with GAN-ZT showing a slight decline. Once again Go-ZT outperformed all other models. Go-ZT produced an SE, SP, and PPV of 71.4%, 91.8%, and 55.6% respectively. GAN-ZT on the other hand produced SE, SP, and PPV values of 71.4%, 59.2%, and 20.0%, respectively. Evaluation of the chemical domain space using Go-ZT and GAN-ZT showed that chemicals should be excluded due to long chain length, and betaine or Chloroperfluoro functional groups as these chemical properties fall outside of the domain space of our models. The results show that Go-ZT performed best with increases in SE, PPV, Kappa, and AUROC values while GAN-ZT saw declines in PPV, and Kappa values (Fig 7).

**Table 3:** Performances of different methods in activity prediction of test set chemicals.

Model	SE	SP	PPV	Kappa	AUROC
SVM	28.6	95.9	50.0	0.300	0.649
MLP	28.6	89.8	28.6	0.184	0.660
RF	28.6	98.0	66.7	0.351	0.459
GAN-ZT	71.4	59.2	20.0	0.146	0.653
Go-ZT	71.4	91.8	55.6	**0.564**	**0.816**

### Combined model

**Fig 8:**
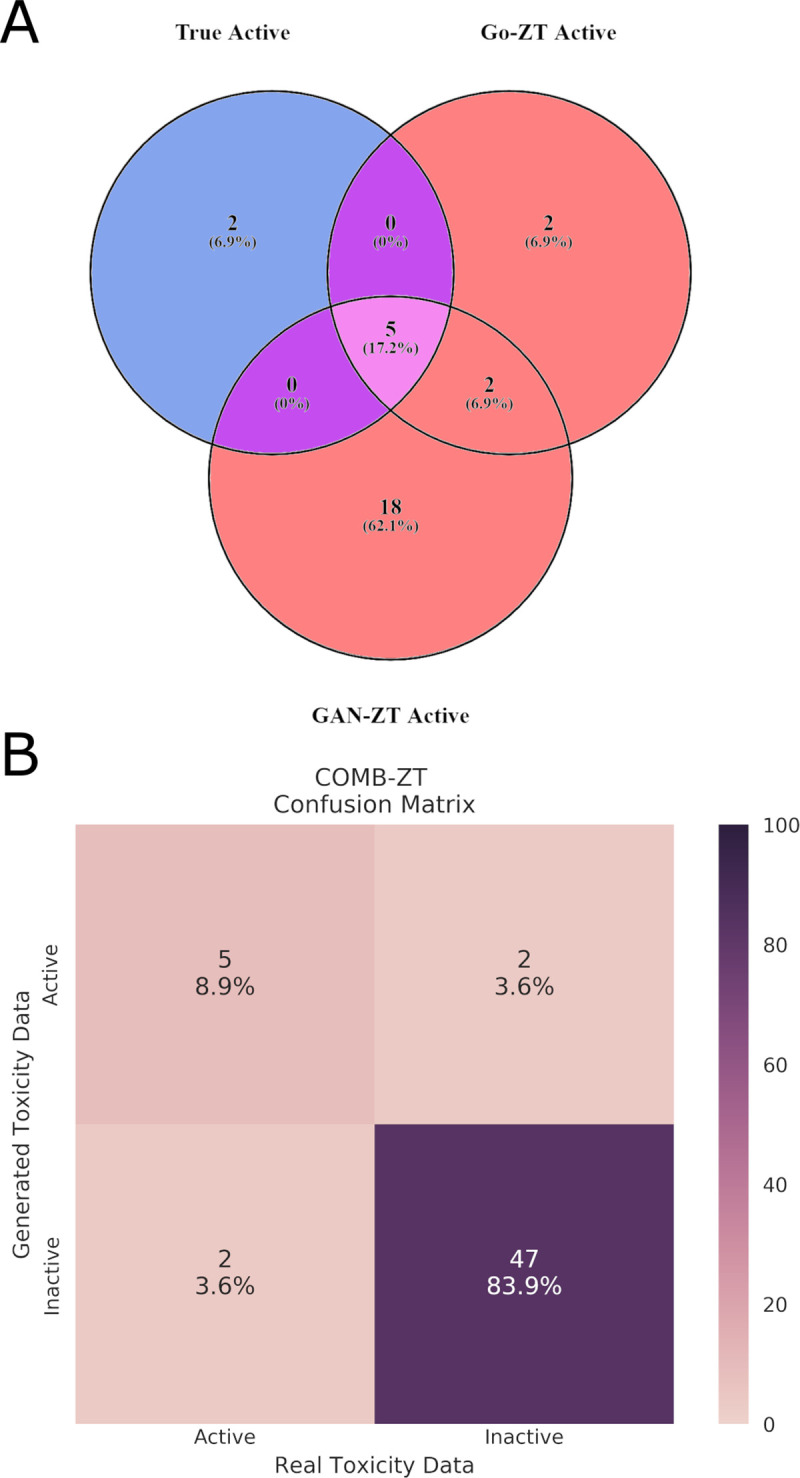
Model consensus on chemical activity. (A)Venn diagram showing the overlap between true active chemicals and chemicals predicted to be active by either Go-ZT or GAN-ZT. (B) A confusion matrix showing the performance of the combined Go-ZT and GAN-ZT models using the test dataset.

Model combinations between Go-ZT, and GAN-ZT, SVM, MLP, or RF were assessed for improvement with particular focus on PPV, Kappa, and AUROC. By combining the predictive results of Go-ZT and GAN-ZT we were able to improve the SP, Kappa, and AUROC to 95.9%, 0.673, and 0.837, respectively (Fig 8). As a result of the consensus between the models we were able to capture five of the seven active chemicals in the test set while eliminating two false positives which translates to a PPV of 71.4%.

### Random label shuffling shows no predictive power

We performed 1000 random shuffling’s to construct 1000 label shuffled test datasets for analysis using our models. Table 4 shows that the models performed poorly. The poor performance indicated that random noise is unlikely driving model performance.

**Table 4:** Model performance using shuffled data.

Model	Kappa		AUROC	
	Mean	SD	Mean	SD
GAN-ZT	0.004	0.10	0.504	0.10
Go-ZT	0.021	0.13	0.513	0.08

## Discussion

GAN-ZT and Go-ZT architecture with chemical structure vectorization using views predicts empirical toxicity results with fair to good Kappa values of 0.160 and 0.495, respectively. GAN-ZT, SVM, RF, and MLP models performed similarly on the training dataset while the SVM and RF performed better than the GAN-ZT and MLP on the test dataset. Go-ZT significantly outperformed all models on both the training and test datasets. Go-ZT predicted active chemicals with an SE of 71.4%, SP of 91.8%, and a PPV of 55.6% while GAN -ZT predicted active chemicals with an SE of 71.4%, SP of 59.2%, and a PPV of 20.0%. When we examined the overlap in predicted active chemicals between Go-ZT and the other four models only in combination with GAN-ZT did we see that a consensus model improved the SP, PPV, Kappa, and AUROC to 95.9%, 71.4%, 0.673, and 0.837, respectively. These results show that a regression-based DNN is capable of predicting toxic developmental activity with good efficacy in both the training and test datasets. Further, by leveraging the strengths of both the supervised generative adversarial network and DNN the intersection between the models was able to accurately predict the toxicity of chemicals not part of the initial ToxCast screen.

A wide range of machine learning methods such as Deep Neural Networks (DNN), Support Vector Machines (SVM), *k*-nearest neighbor (*k*-NN), gradient-boosted decision trees, and Bayesian Classifiers have been applied to cheminformatics problems to predict biologically active chemicals with AUROC values ranging from 0.7–0.83 [62]. These studies utilized molecular fingerprints of chemicals from the ChEMBL database, and single biological activity prediction in drug discovery but were not focused on *in vivo* toxicity. More recent studies have used data from the Tox21 database as part of the NIH 2014 Tox21 Data Challenge with multitask DNNs outperforming other machine learning methods with AUROC values ranging from 0.69–0.92 in 12 different biochemical assays [44]. Further, Mansouri and Judson successfully built a QSARs model for G-protein coupled receptor assays using partial least square discriminant analysis that resulted in a balanced accuracy of 96%[63]. Our combined model produced a similar AUROC and a lower balanced accuracy value (83.7%). To the best of our knowledge, this is the first study to develop a DNN model without explicit use of molecular descriptors to predict *in vivo* toxicity in a large chemical set. While our model is a potentially useful tool for prioritizing chemicals for screening tests, it does have its limitations including chemical domain space, and our networks are dependent on accurate 3D chemical structural information to produce reliable results and at this time are not designed to evaluate mixtures.

Overall, our results show that a DNN utilizing 3D chemical structural information is a useful prescreening tool for predicting the toxic outcomes of the approximately 80,000 untested chemicals registered with the EPA. If we consider that between the training, and test datasets there are 1,059 chemicals and only 166 are active (15.7%) then there are possibly 12,540 untested active chemicals registered. If the PPV of the combined model holds at 71.4% this would result in a list of ~17,500 chemicals to screen. While still a very large number it would reduce the experimental space by over three-quarters. Further, these compounds may then be ranked by AggE and the chemicals with the highest ranking identified should then be prioritized for assessment using the zebrafish HTS assay for developmental toxicity, as these assays are considerably faster and cheaper than traditional chemical screens in mammalian systems.

Looking to the future, increasing computational resources and chemical structural data, alternative network architectures, inclusion of ToxCast assay results, and zebrafish behavioral endpoints in conditional training could improve the predictive value of DNN in *in vivo* toxicity testing. The views chemical vectorization methodology needs to be further evaluated with existing machine learning algorithms. There is also potential to add other chemical information to the views methodology including charge and types of bonds. Additional work needs to be done to assess the utility of cGANs as a tool to evaluate structure activity relationships (SAR) in *in vitro* toxicology and finally DNNs need to be adapted to evaluate mixtures if a sufficiently large dataset is available.
